# TIR-Domain-Containing Adapter-Inducing Interferon-β (TRIF) Is Essential for MPTP-Induced Dopaminergic Neuroprotection via Microglial Cell M1/M2 Modulation

**DOI:** 10.3389/fncel.2017.00035

**Published:** 2017-02-22

**Authors:** Minghui Shan, Sen Lin, Shurong Li, Yuchen Du, Haixia Zhao, Huarong Hong, Ming Yang, Xi Yang, Yongmei Wu, Liyi Ren, Jiali Peng, Jing Sun, Hongli Zhou, Bingyin Su

**Affiliations:** ^1^Development and Regeneration Key Lab of Sichuan Province, Department of Pathology, Department of Anatomy and Histology and Embryology, Chengdu Medical CollegeChengdu, China; ^2^Department of Clinical Pathology, Nanyang Central HospitalNangyang, China; ^3^Research Center, Chengdu Medical College Infertility HospitalChengdu, China

**Keywords:** TRIF, microglia, M1/M2, Parkinson's disease, neuronal apoptosis

## Abstract

Dynamic changes of two phenotypes of microglia, M1 and M2, are critically associated with the neurodegeneration of Parkinson's disease. However, the regulation of the M1/M2 paradigm is still unclear. In the MPTP induced neurodegeneration model, we examined the concentration of dopamine (DA) related metabolites and the survival of tyrosine hydroxylase (TH) positive cells in WT and *Trif* ^−/−^ mice. In *in vitro* experiments, MN9D cells were co-cultured with BV2 cells to mimic the animal experiments. Inhibition of TRIF aggravated TH+ cell loss, and DA-related metabolites decreased. TRIF inhibition was able to interrupt the microglial M1/M2 dynamic transformation. More BV2 cells were activated and migrated across the membrane of transwell plates by siTRIF treatment. Also, TRIF interruption inhibits the transformation of BV2 cells from the M1 to M2 phenotype which played a beneficial role in neuronal degenerative processes, and increased MN9D apoptosis. Moreover, MPP+ treatment decreases the (DAT) dopamine transporter and TH synthesis by MN9D. Taken together, the current results suggest that TRIF plays a key switch function in contributing to the microglial M1/M2 phenotype dynamic transformation. The interruption of TRIF may decrease the survival of MN9D cells as well as DAT and TH protein production. The current study sheds some light on the PD mechanism research by innate inflammation regulation.

## Introduction

Parkinson's disease (PD) is a neurodegenerative disorder of the central nervous system that affects some people above the age of 60 worldwide. The pathological changes of PD include the death of the nigrostriatal dopaminergic midbrain neurons, a significant reduction of dopamine in the striatum and the presence of eosinophilic inclusion bodies in the surviving neurons in the substantia nigra (Dauer and Przedborski, 2003). Neuroinflammation is a prominent event that affects the clinical course of Parkinson's disease (Gao and Hong, 2008; Glass et al., 2010). During neurodegenerative processes, microglia plays a crucial role in regulating CNS inflammation. Molecular and clinical evidence has shown significant increase of microglial activation, accumulation, and concentration of inflammatory factors in pathological neurodegenerative diseases (LaVoie et al., 2004; Ouchi et al., 2005; Gerhard et al., 2006; Glass et al., 2010). In PD patients the presence of persistent microglia activated and accumulated in the vicinity of the neurons in substantia nigra (Ferrari and Tarelli, 2011; Barcia et al., 2013) has been shown. Although the mechanism how microglia maintains the activation phenotype is still unclear based on current evidence, it has been suggested that microglia activation may contribute to the degeneration of dopaminergic neurons (Barcia et al., 2013).

Neuroinflammation, which is a side effect of the persistent activation of microglia, recently received attention as double-edged sword that executes either prejudicial or beneficial effects on the neurons (Doring and Yong, 2011; Cherry et al., 2014). Its activation can be classified into two major phenotypes which are known as the M1 phenotype (classical activation) and M2 phenotype (alternative activation; Hanisch and Kettenmann, 2007; Colton, 2009). In the M1 phenotype, the classical reactivated phenotypes are associated with iNOS and NF-κB signaling pathway activation, synthesis and release of pro-inflammatory factors such as tumor necrosis factor (TNF)-α, interleukin (IL)-1β, reactive oxygen species (ROS), and nitric oxide (NO) (Le et al., 2001; Block et al., 2007). The M2 phenotype is defined by both alterative and acquired deactivations, which promote phagocytosis of neuron debris and misfolded proteins, tissue repair, extra cellular matrix (ECM) reconstruction, anti-inflammatory antagonized immunosuppression and neural protection associated with IL-10 and transforming growth factor (TGF)-β insult (Colton, 2009; Colton and Wilcock, 2010).

According to our previous study of the activation phenotype of microglia in optic nerve regeneration and intracerebral hemorrhage induced neural inflammation, the Toll-like receptor (TLR) signaling pathway plays a private role in regulating microglial activation and neuroinflammation (Lin et al., 2012a,b). TLR3 and TLR4 signaling pathways are all involved in regulating microglial activation via the release of TRIF and MyD88 adaptor mediated downstream pro-inflammatory factors. TRIF-dependent inflammatory activation, including IRF3 phosphorylation, pro-inflammatory cytokine synthesis and release, the activation of apoptosis-associated mediator Fas, and a decreased number of profitable M2-like CD11b^+^ microglia (Stridh et al., 2013) suggests that the TLR3/TRIF signaling pathway may play a crucial role in regulating microglia induced neuroinflammation and in the microglial M1/M2 paradigm. However, the TLR3-TRIF signaling pathway assumes a protective role against West Nile virus in brain tissue (Daffis et al., 2008) and a protective role in retinal pigmented epithelium (RPE) against oxidative stress (Patel and Hackam, 2013). Thus, TLR3-TRIF signaling in different tissues and conditions may have a detrimental or a beneficial effect.

In addition, there is no such evidence that indicates the microglial M1/M2 polarization regulation by TLR3/TRIF in PD. In this study, we investigate the role of TRIF in regulating the transformation of the microglial M1/M2 phenotype in the mesencephalon-derived dopaminergic neuronal cell line (MN9D) and demonstrate that microglial TRIF plays an important role in regulating MN9D cell survival and microglial M1/M2 modulation.

## Materials and methods

### Animals and 1-methyl-4-phenyl-1,2,3,6-tetrahydropyridine (MPTP) induced neural degeneration

Male C57BL/6 mice (8–12 week old, 20–24 g, from Scientific Research Center, Chengdu Medical College, Chengdu, China), and male adult *Trif* ^−/−^ mice (C57BL/6 J-AW046014 Lps2 /J; from Jackson Laboratory, Bar Harbor, ME, USA, gifts from Prof. Qingwu Yang, Xinqiao Hospital, Third Military Medical University, Chongqing, China), age 8–12 weeks (20–24 g). All animal-related procedures in this study were performed according to the Chengdu Medical College guidelines for the care and use of experimental animals. The Animal Ethics Committee of Chengdu Medical College approved all animal experimental procedures used in the present study, which are in accordance with the principles outlined in the National Institute of Health (NIH) Guide for the Care and Use of Laboratory Animals. MPTP (Sigma-Aldrich, Shanghai, China) was freshly dissolved in 0.9% saline and administered to mice intraperitoneally (i.p. 20 mg/kg) four times within a 2 h interval.

### Chemicals and biological reagents

Methyl-4-phenyl tetrahydropteridine (MPP+ iodide), MPTP and lipopolysaccharide (LPS) were purchased from Sigma (Shanghai, China). Recombinant Mouse IL-4 was purchased from R&D Systems (Shanghai, China). Poly(I:C) was purchased from InvivoGen (CA, USA).

### MN9D and BV2 cell culture

The MN9D dopaminergic cell line and the immortalized murine BV2 microglial cell line were generous gifts by Prof. Qun-Yuan Xu, Capital Medical University, Beijing, China. These cells were cultured at 37°C plus 5% CO_2_ in a high glucose (4500 mg/L) Dulbecco's Modified Eagle Medium (Gibco, Shanghai, China) supplemented with 10% heat-inactivated fetal bovine serum (Gibco, Shanghai, China) and 1% streptomycin and penicillin (Life Technologies, Shanghai, China). For all experiments, MN9D cells were differentiated for 4 days with 1.5 mM sodium butyrate (Sigma, Shanghai, China). All methods were carried out in line with relevant guidelines and regulations of Chengdu Medical College.

### BV2 cell M1/M2 paradigm

The BV2 cells were treated with LPS (100 ng/mL) or IL-4 (20 ng/mL) to induce polarity of BV2 cells followed by real-time RT PCR identification. Then, the cells were collected from the culture plates by using a rubber policeman and centrifuged at 300 × g for 5 min, washed with ice-cold PBS twice followed by TRIzol® (Life Technologies, Shanghai, China) lysis. RNA was collected by the TRIzol® lysis protocol according to the manufacturer's protocol.

### Quantitative real-time-PCR

After treatment total RNA was extracted from the BV2 cells with TRIzol® reagent (Life Technologies, Shanghai, China) according to the manufacturer's protocol. Total RNA was used for cDNA synthesis with the PrimeScript™ RT reagent Kit (TaKaRa, Dalian, China). SYBR™ Green quantitative PCR was performed with validated primers such as IFN-β, TNF-α, IL-6, CD86, inducible NO synthase (iNOS), IL-10, CD206, Arginase1, Ym1, and β-actin (Life Technologies, Shanghai, China) with SYBR™ premix Ex Taq™ II kit (Takara, Dalian, China) and monitored by a IQ5 Real-time PCR machine (Bio-Rad, CA, USA). The relative expression levels of each mRNA were calculated by using the 2^−ΔΔCt^ algorithm normalizing to β-actin and relative to the control samples.

### High performance liquid chromatography with electrochemical detection (HPLC-EC) analysis of DA and related metabolites in striatum (Str) in WT and *Trif*^−/−^ mice

MPTP treated WT and *Trif* ^−/−^ mice were sacrificed by CO_2_ asphyxiation on the Day 7 after the last MPTP injection according to the method described previously (Liang et al., 2007). Briefly, the Str tissue of brains were dissected out on ice immediately. Dissected Str tissues were homogenized in 50 μl of 0.1 M perchloric acid. After centrifugation (15,000 × g, 10 min, 4°C) and filtration, 30 μl supernatant was injected onto a C18 reverse-phase HR-80 catecholamine column (ESA, Bedford, MA, USA). The concentrations of dopamine (DA) and its metabolites 3,4-dihydroxyphenylacetic acid (DOPAC), norepinephrine (HE), 5-hydroxyindoleacetic acid (5-HIAA), 5-hydroxytryptamine (5-HT), and homovanillic acid (HVA) were quantified by HPLC-EC detection. The mobile phase (pH 2.9) consisted of 275 mg/l octane sulfonic acid in 90% 75 mM sodium phosphate and 10% methanol; the flow rate was 1 mL/min. Peaks were detected by an ESA Coulochem II with a model 5010 detector (E1 = 50 mV, E2 = 400 mV). Data were collected and processed by a Chromeleon™ computer system (Gynkotek, Gemering, Germany).

### Conditioned medium collection and MN9D cell induction

Conditioned Medium (CM) was collected at desired time points after cell treatment, filtered through 0.45 μm filters (Millipore, Shanghai, China) and quickly frozen at −80°C for further MN9D cell culture. MN9D cells grown on poly-D lysine (Sigma, Shanghai, China)-coated slides were pretreated with MPP+ (300μM) for 12 h after which time the medium was removed, and cells were exposed to conditioned medium from BV2 cells for 24 h.

### siRNA transfection

BV2 cells were plated in 6-well-tissue culture plates at about 80% confluence. The TRIF siRNA (5′-GGGUUACCACACGAAAUUAtt-3′, 5′-GCCUCUCAUUAUUCACCAUtt-3′) and scrambled negative control siRNA (Cat. 4390843, Life Technologies, Shanghai, China) were obtained from Life Technologies. Transfections were performed using Lipofectamine™ 2000 (Life Technologies, Shanghai, China) according to the manufacturer's instructions. Four to six hours after transfection, media were removed and replaced with fresh media. Cells were treated with poly(I:C) (25 μg/mL) and vehicle for further experiments on the following day.

### Transwell migration assay

BV2 cells are placed on the upper layer of a cell permeable membrane and MN9D cells were placed on the bottom of the cell culture plate. Following a culture period of 24 h, the BV2 cells that have migrated through the membrane are quantified and stained by cresyl violet.

### Immunoblotting

Total protein from each group was digested in radioimmunoprecipitation (RIPA) assay buffer supplemented with a protease inhibitor cocktail (Roche, Shanghai, China). Supernatant was collected after homogenate centrifugation at 12,000 g for 10 min at 4°C. After protein denaturation, the proteins were separated by 10% sodium dodecyl sulfate polyacrylamide gel electrophoresis, and the resolved proteins were transferred onto polyvinylidene difluoride membranes (Bio-Rad, CA, USA). Each membrane was blocked and then incubated with primary antibody at 4°C overnight followed by incubation with HRP-conjugated secondary antibodies for 1 h at 25°C. The intensity of the protein signal of three duplicates from each sample was calculated using ImageJ software (NIH, USA). The antibodies used were anti-TLR3 (1:1,000, ab62566, Abcam, Cambridge, UK), anti-TRIF (1:1,000, ab13810, Abcam, Cambridge, UK), anti-IRF3 (1:1,000, sc-9082, Santa Cruz Biotechnology, CA, USA), anti-phospho-IRF3 (1:1,000, 4,947, Cell Signaling Technology, MA, USA), anti-TH (1:2,000, AB152, Millipore, MA, USA), and anti-DAT (1:1,000, ab111468, Abcam, Cambridge, UK). Anti-mouse HRP (1:10,000, ZB2305, Zhongshan Goldenbridge, Beijing, China) and anti-rabbit HRP (1:10,000, ZB-2301, Zhongshan Goldenbridge, Beijing, China) were used as secondary antibodies. Anti-glyceraldehyde-3-phosphate dehydrogenase (GAPDH, 1:5,000, KC-5G4, KANGCHEN) served as a loading control.

### Immunocytochemistry and tunel staining

The CM-treated cells were washed with icecold PBS twice followed by 4% paraformaldehyde fixation (PFA, Sigma, Shanghai, China) for 20 min. TUNEL (Shanghai, China) staining was performed according to manufacturer's instructions. Briefly, the cells were permeabilized with 0.1% Triton™ X-100 and 0.1% sodium citrate for 2 min on ice followed by a PBS rinse. The TUNEL mixture was prepared freshly and added to each sample for incubation at 37°C for 1 h in the dark. The samples were rinsed twice with PBS, then blocked with 5% bovine serum albumin (0.05% Tween® 20) in PBS for 45 min at room temperature. Cells were then incubated with primary antibody anti-βIII tubulin (1:400, ab78078, Abcam, Cambridge, UK) at 4°C overnight. Appropriate secondary antibodies (Alexa Fluor® 568, Life Technologies; A11031, 1:400) were used for incubation at 25°C for 1 h. After washing with PBS, nuclei were counterstained with 4′,6-diamidino-2-phenylindole (DAPI; 1 μg/ml) for 5 min. Cells were mounted with an inverted fluorescent mounting medium (DAKO, Glostrup, Denmark), and images were captured with a digital camera (Sterling Heights, MI).

### TH immunostaining

Naïve, MPTP, MPTP+poly(I:C) and poly(I:C) treated mice were anesthetized by chloral hydrate (4%) and were fixed by 2% PFA perfusion through the heart. After 10, 20, and 30% sucrose dehydration, the fixed brains were then sectioned in a cryostat to get the SNc sections (30 μm). After PBS rinsing three times (5 min each), the sections were treated with 0.3% H_2_O_2_ for 25 min at room temperature (r.t.). After PBS rinsing, the sections were blocked in goat serum (5%) for 1 h at r.t., and primary antibody (TH, 1:2,000; Millipore, CA, USA) was added for incubation overnight at 4°C. On Day 2, following PBS rinsing, biotinylated goat anti-rabbit secondary antibody was added onto the slides which were incubated for 1 h, followed by incubation with Streptavidin-HRP for 1 h at r.t. and visualization by reaction with nickel-intensified, 3-diaminobenzidine tetrahydrochloride (DAB: 0.25% nickel ammonium sulfate/0.05% DAB as a chromagen and 0.003% hydrogen peroxide) for 5 min. Sections were mounted on gelatin-coated slides, dehydrated through graded ethanol, and cleared in xylene and finally followed by coverslipping using Permount. The images were taken by an Olympus microscope (Japan).

### Stereological counting of TH-immunoreactive neurons

Based on our previous quantification method and protocol (Liang et al., 2006, 2007, 2008), density of TH-immunopositive cells was counted on both hemispheres by serial section analysis of the total number of neurons. Briefly, according to the atlas of mouse brain (The Mouse Brain in Stereotaxic Coordinates, Academic Press, New York, 2001), every sixth brain section throughout the entire extent of the SNc was numbered from the rostral to the caudal plane by blinded investigators (Crocker et al., 2003). Adjacent SNpc tissue sections from each animal were also stained with methylene blue (Nissl's staining) to validate immunohistochemical determination of nigral neuron survival. TH positive cells were quantified in SNpc tissue sections from B-2.8 to B-3.52 according to our previous method (Liang et al., 2006). Estimate of total TH positive neuron populations was calculated using Abercrombie's correction (Abercrombie, 1946). The images of the SNc region were taken by a BX63 microscope (Olympus, Japan). The data output is shown as number of TH positive cells.

### Flow cytometry analysis

The occurrence of apoptosis was determined by the fluorescein isothiocyanate (FITC) annexin V Apoptosis Detection Kit (Keygen, Nanjing, China) after cell treatment with MPP+ (300 μM) and BV2-conditioned medium for 24 h using a C6 flow cytometer (BD Biosciences, NJ, USA).

### Statistical analysis

All data shown are mean ± S.E.M of triplicate values from three separate experiments. ^*^p < 0.05, ^**^p < 0.01, ^***^p < 0.001 were indicated as compared with the control group. Independent Student *t*-test or one-way ANOVA was used to compare the continuous variables between the two groups or more than two groups. Statistical analysis was carried out with statistical analysis software program SPSS13.0 software (IBM) and Prism® 5.0 software (GraphPad).

## Results

### High performance liquid chromatography with electrochemical detection (HPLC-EC) analysis of DA-related metabolites in Str between WT and *Trif*^−/−^ mice

**Figure 1:**
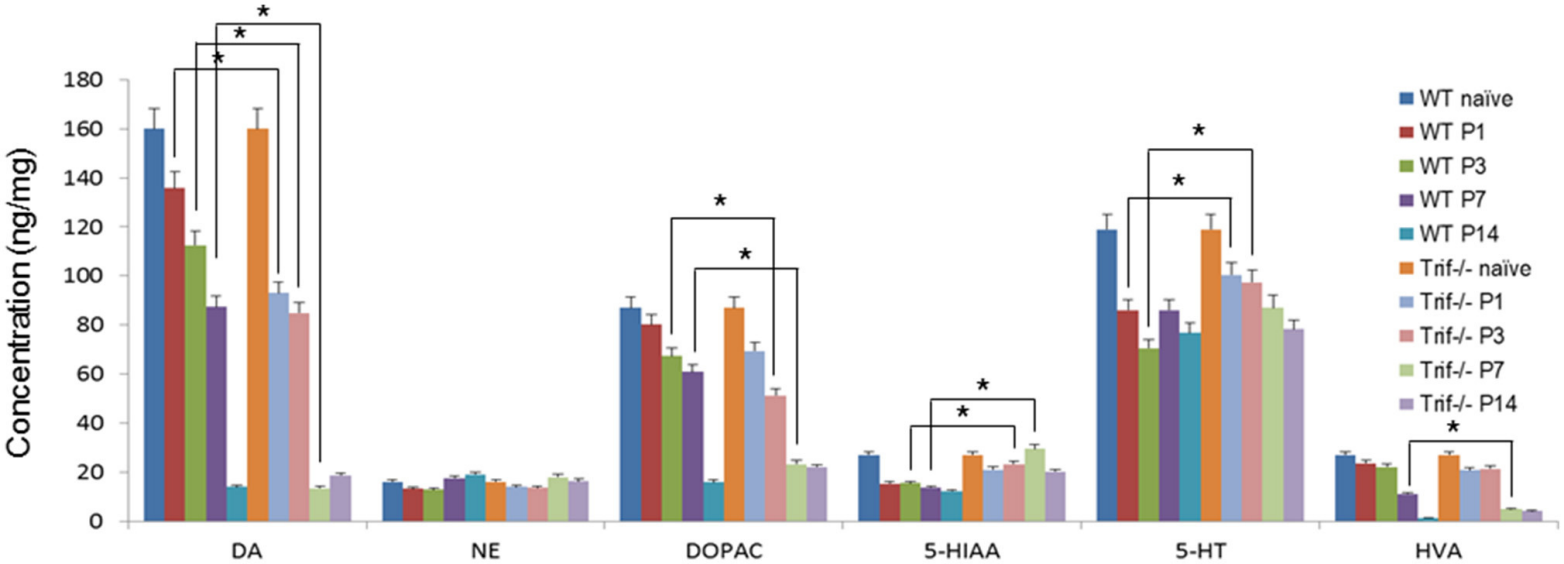
**Concentrations of dopamine-related products in Str of MPTP-treated WT and *Trif*^−/−^ mice by HPLC detection**. The concentrations of dopamine, DOPAC, 5-HIAA, 5-HT, and HVA decreased dramatically in both the WT and *Trif* ^−/−^ groups from D1 to D14 post-MPTP treatment (P1–P14). Significant differences of dopamine concentrations were found between WT and *Trif* ^−/−^ groups on P1 (135.1 ± 6.2 vs. 91.8 ± 4.1), P3 (112 ± 4.8 vs. 82.7 ± 2.9), and P7 (84.1 ± 3.5 vs. 16.8 ± 1.8), respectively. And the concentrations of DOPAC decreased significantly in the *Trif* ^−/−^ group compared with the WT group on P3 (67.4 ± 2.5 vs. 51 ± 1.7) and P7 (67.4 ± 2.5 vs. 21.3 ± 1.9) post-MPTP treatment. Moreover, the concentrations of 5-HT are significantly different between WT and *Trif* ^−/−^ groups (^*^p < 0.05, *n* = 6).

DA and its metabolites DOPAC, HE, 5-HIAA, 5-HT, and HVA were detected at Day 1, 3, 7, and 14 after MPTP injection in WT and *Trif* ^−/−^ mice (Figure 1). MPTP significantly reduced DA levels, DOPAC levels and 5-HT levels in WT and *Trif* ^−/−^ mice from Day D1 to D14 post-MPTP treatment, respectively. Interestingly, the levels of DA (D1, D3, and D7), DOPAC (D3 and D7), 5-HIAA (D3 and D7), 5-HT (D1 and D3), and HVA (D7) were dramatically decreased in *Trif* ^−/−^ mice compared to respective levels in WT mice (Figure 1, *p* < 0.05, *n* = 6). However, no effect on the genotype was found with respect to the NE level.

### TRIF deficiency deteriorates MPTP-induced DA neuron loss

**Figure 2:**
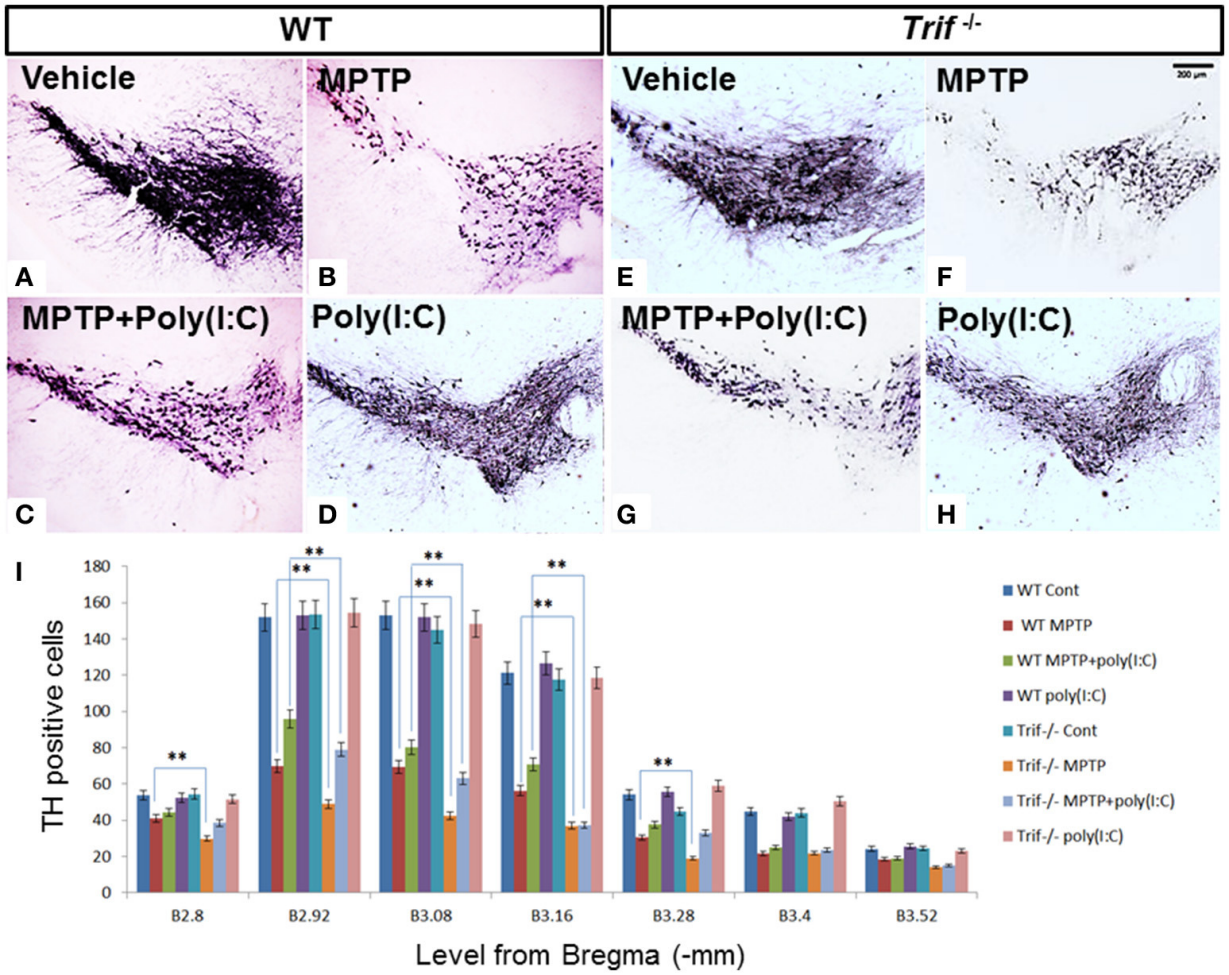
**TH staining of SNpc in WT and *Trif*^−/−^ mice at D7 with or without poly(I:C) treatment. (A–D)** Representative microphotographs of TH-positive cells in SNc of WT mice. **(E–H)** Representative microphotographs of TH-positive cells in SNc of *Trif* ^−/−^ mice on D7. **(I)** Quantification of TH-positive neurons in SNc in WT and *Trif* ^−/−^ mice. More TH-positive neurons were found in MPTP-treated WT mice than that in *Trif* ^−/−^ mice in B2.8 (41 ± 3.0 vs. 31 ± 2.2), B2.92 (69.8 ± 5.2 vs. 48.8 ± 3.2), B3.08 (69.4 ± 4.1 vs. 42.4 ± 3.2), B3.16 (56.2 ± 3.6 vs. 36.8 ± 2.4), and B3.28 (30.4 ± 1.8 vs. 19 ± 2.0) groups. More TH-positive neurons were found in the MPTP+poly(I:C) treated WT group than that in *Trif* ^−/−^ group in B2.92 (69.8 ± 5.2 vs. 48.8 ± 3.2), B3.08 (69.8 ± 5.2 vs. 48.8 ± 3.2), and B3.16 (69.8 ± 5.2 vs. 48.8 ± 3.2) levels. Number of TH positive neurons were significantly decreased in both WT and *Trif* ^−/−^ mice when MPTP and MPTP+poly(I:C) treated, respectively (WT MPTP vs. WT Cont, ^**^p < 0.01; WT MPTP+poly(I:C) vs. WT Cont, *p* < 0.01; *Trif* ^−/−^ MPTP vs. *Trif* ^−/−^ Cont, ^**^p < 0.01; *Trif* ^−/−^ MPTP+poly(I:C) vs. *Trif* ^−/−^ Cont, *p* < 0.01; *n* = 6).

To confirm the effect of an MPTP-induced decrease of DA and related metabolites, we then observed dopamine neuron loss by tyrosine hydroxylase (TH) staining in SNpc by quantification in serial sections from level (from Bregma, −2.8 to 3.52 mm) B2.8 to B3.52. A decreased number of these cells were clearly observed in the MPTP treatment group in WT and *Trif* ^−/−^ mice on D7 from B2.8 to B3.52 levels. However, less TH positive cells were found in MPTP treated *Trif* ^−/−^ mice (Figures 2F,I) vs. the WT group in B2.8, B2.92, B3.08, B3.16, and B3.28 levels (Figures 2B,I, *p* < 0.05, *n* = 6), which suggests that TRIF may play a protecting role in the neuronal MPTP-induced DA neuron loss. To verify the function of the TRIF signaling pathway in DA neuron loss, we used poly(I:C), an agonist of the TLR3-TRIF signaling pathway, to rescue the neuronal loss phenotype in MPTP-treated WT and *Trif* ^−/−^ mice at a concentration of 1.25 μg/g dose via i.p. injection. By poly(I:C) treatment, more TH-positive cells survived in the WT group (Figure 2C) compared with *Trif* ^−/−^ mice (Figure 2G) in B2.92, B3.08, and B3.16 levels (Figure 2I, *P* < 0.05, *n* = 6), which suggests that the TLR3-TRIF signaling pathway may contribute to DA neuron protection from MPTP-induced neuron loss. No difference in the number of DA neurons was observed between WT and *Trif* ^−/−^ mice in the poly(I:C) treatment group (Figures 2D,H, *p* > 0.05, *n* = 6) as well as in the vehicle group (Figures 2A,E, *p* > 0.05, *n* = 6).

### TRIF-IRF3 signaling pathway can be inhibited by siTRIF in BV2 cells

**Figure 3:**
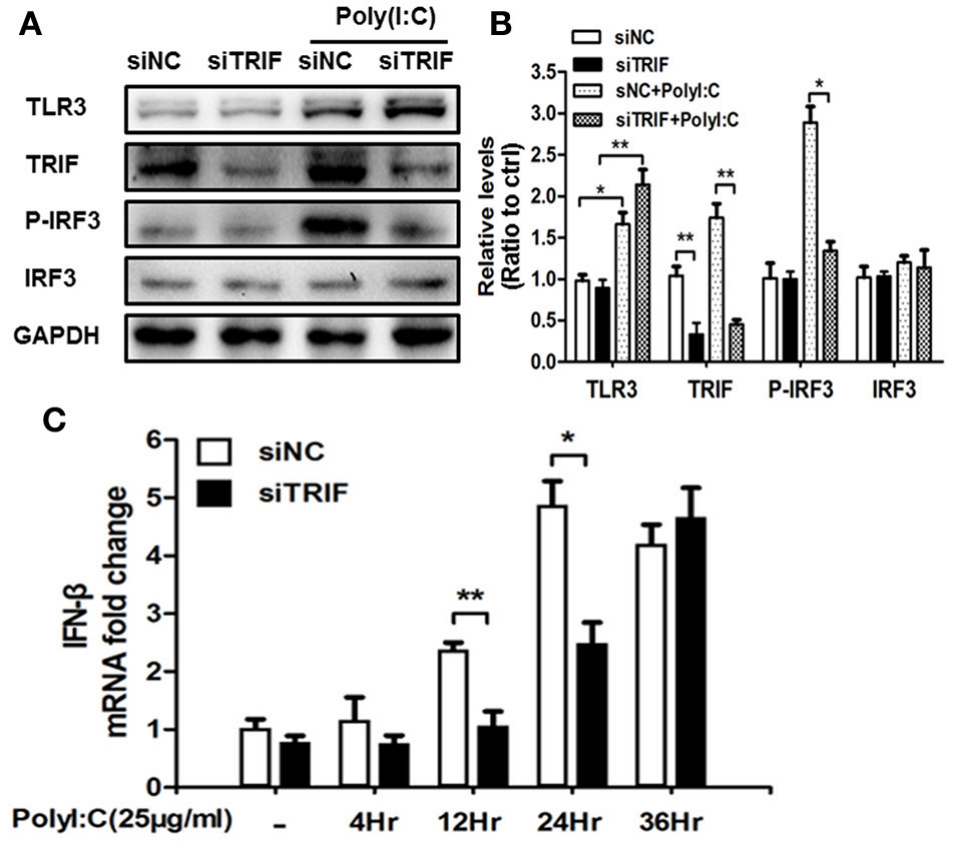
**siTRIF inhibits the expression of TRIF, p-IRF3, IRF3 in BV2 cells**. **(A)** Expression of TLR3, TRIF, p-IRF3, IRF3 in BV2 cells by Western blot detection to verify poly(I:C) stimulation and siRNA inhibition. Reduced levels of TRIF and p-IRF3 were demonstrated in the siTRIF group even with poly(I:C) stimulation. **(B)** Relative expression levels of TLR3, TRIF, p-IRF3, and IRF3 vs. GAPDH expression quantified by software. Reduced relative levels of TRIF and p-IRF3 were quantified in the siTRIF group and poly(I:C) stimulation. *n* = 3, mean ± SEM. ^*^p < 0.05, ^**^p < 0.01. **(C)** Expression of IFN-β mRNA at different time points in BV2 cells stimulated by poly(I:C). *n* = 3, mean ± SEM. ^*^p < 0.05, ^**^p < 0.01.

IRF-3 is one of the downstream molecules of TRIF (Liu et al., 2015), which can be activated by phosphorylation by the inhibitory kappa B kinase (IKK) and/or TANK-binding kinase 1 (TBK1) in response to stimulation (Bruni et al., 2013; Liu et al., 2015). Poly(I:C) is the classic agonist of TLR3, which stimulates TLR3 via a TRIF-dependent pathway which is a unique adaptor in TLR3 and TLR4 signaling pathways contributing to interferon (IFN)-beta production (Yamamoto et al., 2003). To investigate the role of the TLR3-TRIF-IRF3 signaling pathway in BV2 cell stimulation, we treated BV2 cells with siTRIF for 24 h at a concentration of 25 μg/ml and found that the expression of TRIF decreased about 60% compared with the siNC group (Figure 3B, *p* < 0.01) as well as poly(I:C) stimulation group decreased about 70% compared with the siNC group (Figure 3A). Moreover, p-IRF3 which reflects the activation status of the IRF3 signaling pathway also decreased about 50% compared with siNC+poly(I:C) group (Figure 3B, *p* < 0.01) which suggests that the TRIF-IRF3 signaling pathway can be inhibited significantly by siTRIF. The results can be used to set up an inhibition model that will be useful for subsequent experiments.

TRIF, but neither MyD88 nor TIR domain-containing adaptor protein (TIRAP), is able to activate the IFN-β promoter (Yamamoto et al., 2002). The outcome of activation is usually followed by the activation of IFN-inducible genes, such as interferon-inducible protein-10 (IP-10) and glucocorticoid attenuated response gene 16 (GARG16), which were induced in response to LPS in MyD88 knock out cells (Doyle et al., 2002). IFN-β is the typical factor that can be released when the TRIF-IRF3 axis is stimulated (Sharma et al., 2003). We used Real-Time-PCR to identify the change of IFN-β mRNA after siTRIF treatment in the BV2 cell line. The results showed that the gradual upregulation of IFN-β mRNA depends on the time course of the siTRIF treatment. There are significant differences of IFN-β mRNA fold-change between the 12 and 24 h treatment groups, in which the siNC group reached the highest level of mRNA expression at 24 h (Figure 3C, *p* < 0.01). The present results indicate the function of siTRIF on BV2 cells depends on the time course of the siTRIF treatment.

### BV2 cell migration can be attenuated by siTRIF treatment when co-cultured with MPP+-treated MN9D cells

**Figure 4:**
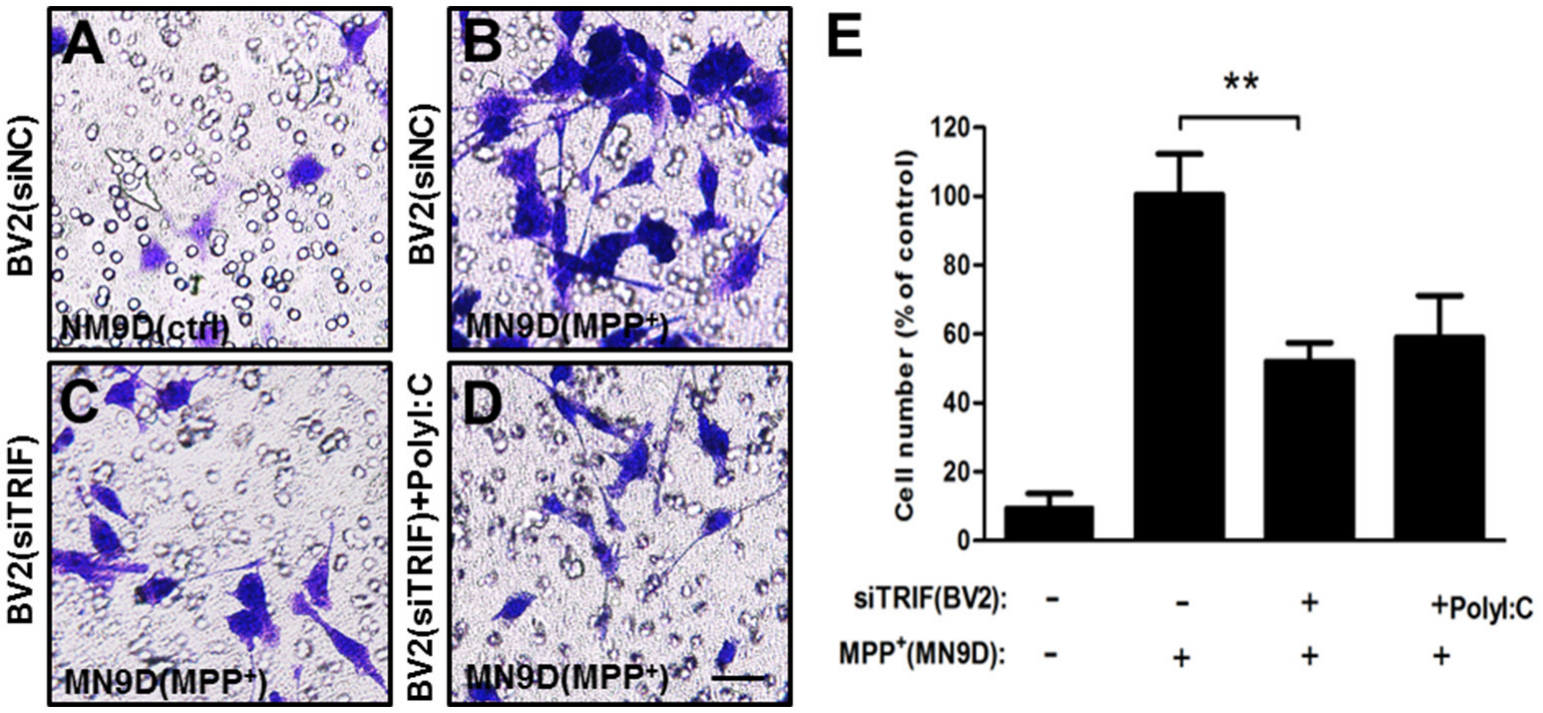
**Migration of activated BV2 cells (top layer of the transwell) in a transwell culture system in co-culture with MN9D cells (bottom layer of the transwell)**. **(A)** Above the transwell membrane: BV2 (siNC); at the bottom of the plate: MN9D (control). **(B)** Above the transwell membrane: BV2 (siNC); at the bottom of plate: MN9D (200 μM MPP+). **(C)** Above the membrane: BV2(siTRIF); at the bottom of the plate: MN9D (200 μM MPP+). **(D)** Above the membrane: BV2 [siTRIF+25 μg/ml poly(I:C)]; at the bottom of the plate: MN9D (200μM MPP+). **(E)** Quantification of the number of BV2 cells that migrated across the transwell membrane. *n* = 5, mean ± SEM, ^**^p < 0.01.

Continuous microglial cell activation and migration are important for the pathogenic processes of Parkinson's disease (Kim et al., 2013; Wang et al., 2015). To investigate the role of TRIF in the microglial migration in response to MPP+ (200 μM) treated MN9D cells, we co-cultured BV2 and MN9D cells in a transwell co-culture system to mimic the pathological process between dopamine neurons and resident microglia that exists in Parkinson's disease. By quantifying the number of BV2 cells that located on the other side of the transwell membrane, we found that the number of migrated BV2 cells decreased about 56.4% when compared with the siNC group. There is no significant difference between siTRIF+ (MPP+ treated MN9D cells) and siTRIF+poly(I:C)+(MPP+ treated MN9D cells) groups, i.e., columns 3 and 4 (Figure 4, *n* = 6, *p* < 0.01).

### Microglial M1/M2 marker paradigm in BV2 cells can be regulated by TRIF inhibition

**Figure 5:**
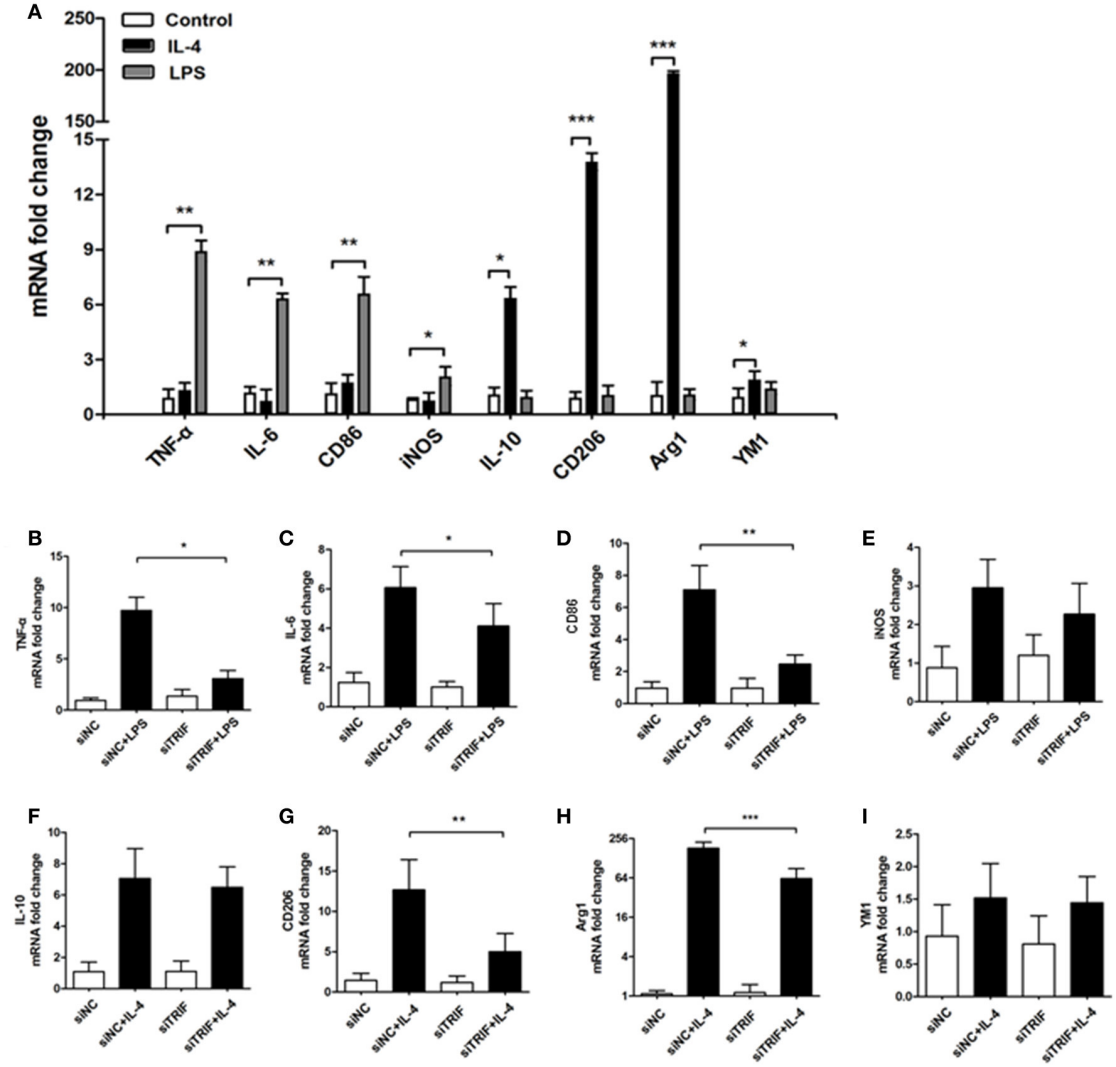
**Inhibition of TRIF contributes to the change of BV2 cell M1/M2 markers stimulated by LPS/IL-4**. **(A)** mRNAs of TNF-α, IL-6, CD86, and iNOs were upregulated significantly by LPS stimulation. mRNAs of IL-10, CD206, Arg1, and YM1 were upregulated significantly by IL-4 stimulation. **(B**–**D,G,H)** Significant differences of mRNA expression of TNF-α, IL-6, CD86, CD206, and Arg1. **(E,F,I)** No difference of mRNA expression between iNOS, IL-10 and YM1. *n* = 3, mean ± SEM, ^*^p < 0.05, ^**^p < 0.01, ^***^p < 0.001.

Lipopolysaccharide (LPS) is known as a classical M1 microglial cell polarization inducer. M1 microglia express pro-inflammatory molecules such as tumor necrosis factor-α (TNF-α), interleukin-1β (IL-1β), interferon-γ (IFN-γ), and nitric oxide (NO). The typical cell surface markers like CD86 and CD68 are also expressed by M1 microglial cells (Liao et al., 2012). On the other hand, IL-4 is known to induce M2 microglial polarization. M2 microglial cells express different molecules, such as IL-4, arignase1, Ym1, CD206, and IL-10, which are beneficial to neuroprotection (Ponomarev et al., 2007; David and Kroner, 2011; Liao et al., 2012). To explore the role of TRIF in the M1/M2 regulation, we added LPS (100 ng/ml) and IL-4 (20 ng/ml) to treat BV2 for 24 h. As indicated by Real-Time PCR results, mRNA expressions of TNF-α, IL-6, CD86, iNOS were upregulated after 24 h LPS treatment, while IL-10, CD206, YM1, and Arg1 were upregulated significantly different from control and LPS treated groups (Figure 5A, *n* = 3, *p* < 0.01). The siTRIF treatment of the different groups resulted in significantly different expressions of TNF-α, IL-6, CD86, CD206, and Arg1 mRNA compared with the control groups (Figures 5B–I, *n* = 3, *p* < 0.01). The results indicated the essential role of TRIF in microglial M1/M2 marker polarization.

**Figure 6:**
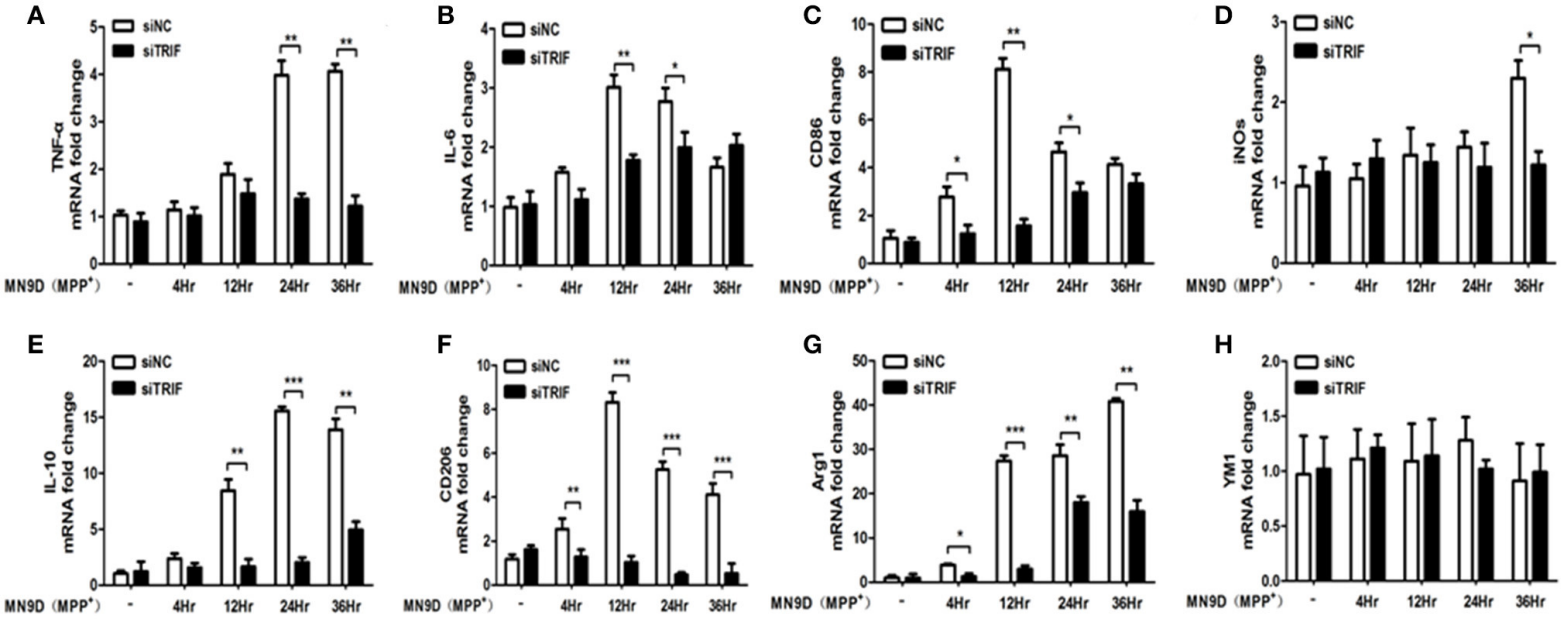
**The effect of MPP+-treated MN9D cells on the M1/M2 marker paradigm of BV2 cells. (A)** Inhibition of the TNF-α mRNA expression in BV2 cells at 24 and 36 h of co-culture with MPP+-treated MN9D cells. **(B)** Inhibition of the IL-6 mRNA expression in BV2 cells at 12 and 24 h of co-culture with MPP+-treated MN9D cells. **(C)** Inhibition of CD86 mRNA expression in BV2 cells at 4, 12, and 36 h of co-culture with MPP+-treated MN9D cells. **(D)** Inhibition of the iNOS mRNA expression in BV2 cells at 36 h of co-culture with MPP+-treated MN9D cells. **(E)** Inhibition of the IL-10 mRNA expression in BV2 cells at 12, 24, and 36 h of co-culture with MPP+-treated MN9D cells. **(F)** Inhibition of the CD206 mRNA expression in BV2 cells at 4, 12, 24, and 36 h of co-culture with MPP+-treated MN9D cells. **(G)** Inhibition of the Arg1 mRNA expression in BV2 cells at 4, 12, 24, and 36 h of co-culture with MPP+-treated MN9D cells. **(H)** No difference of the YM1 mRNA expression in BV2 cells in co-culture with MPP+-treated MN9D cells. *n* = 3, mean ± SEM, ^*^p < 0.05, ^**^p < 0.01, ^***^p < 0.001.

To investigate the stimulating effect of MN9D (MPP+) cells on BV2 cells, which were co-cultured in a transwell system, Real-Time PCR was utilized to quantify the mRNAs of M1/M2 differentiation markers such as IL-1β, IFN-γ, NO, CD86, and CD68. The expressions of M1 markers increased gradually as the co-culture progressed. From 4 to 36 h of co-culture, mRNAs of TNF-α, IL-6, CD86, iNOS, IL-10, CD206, and Arg1 were upregulated gradually. TNF-α and IL-10 reached their highest levels at 24 h, while IL-6, CD86, and CD206 reached their highest levels at 2 h. iNOS and Arg1 reached their highest levels at 36 h. (Figure 6, *p* < 0.01). The present results suggest that the typical microglial M1/M2 markers can be changed by MPP+-treated MN9D cells and the paradigm regulation depends on injured MN9D cell stimulation. Moreover, the inhibition of TRIF had a different effect on the inhibition of mRNA expression levels of typical M1/M2 markers, such as the expression of TNF-α upregulated in 24 and 36 h, IL-6 upregulated from 12 and 24 h, CD86 upregulated from 4 to 24 h, iNOS upregulated in 36 h, IL-10 upregulated from 12 to 36 h, CD206 upregulted from 4 to 36 h and Arg1 upregulted from 4 to 36 h, except for YM1 which has no difference of mRNA fold change from 4 to 36 h (Figures 6A–H).

### Inhibition of TRIF aggravates MPP+-induced MN9D cell apoptosis

**Figure 7:**
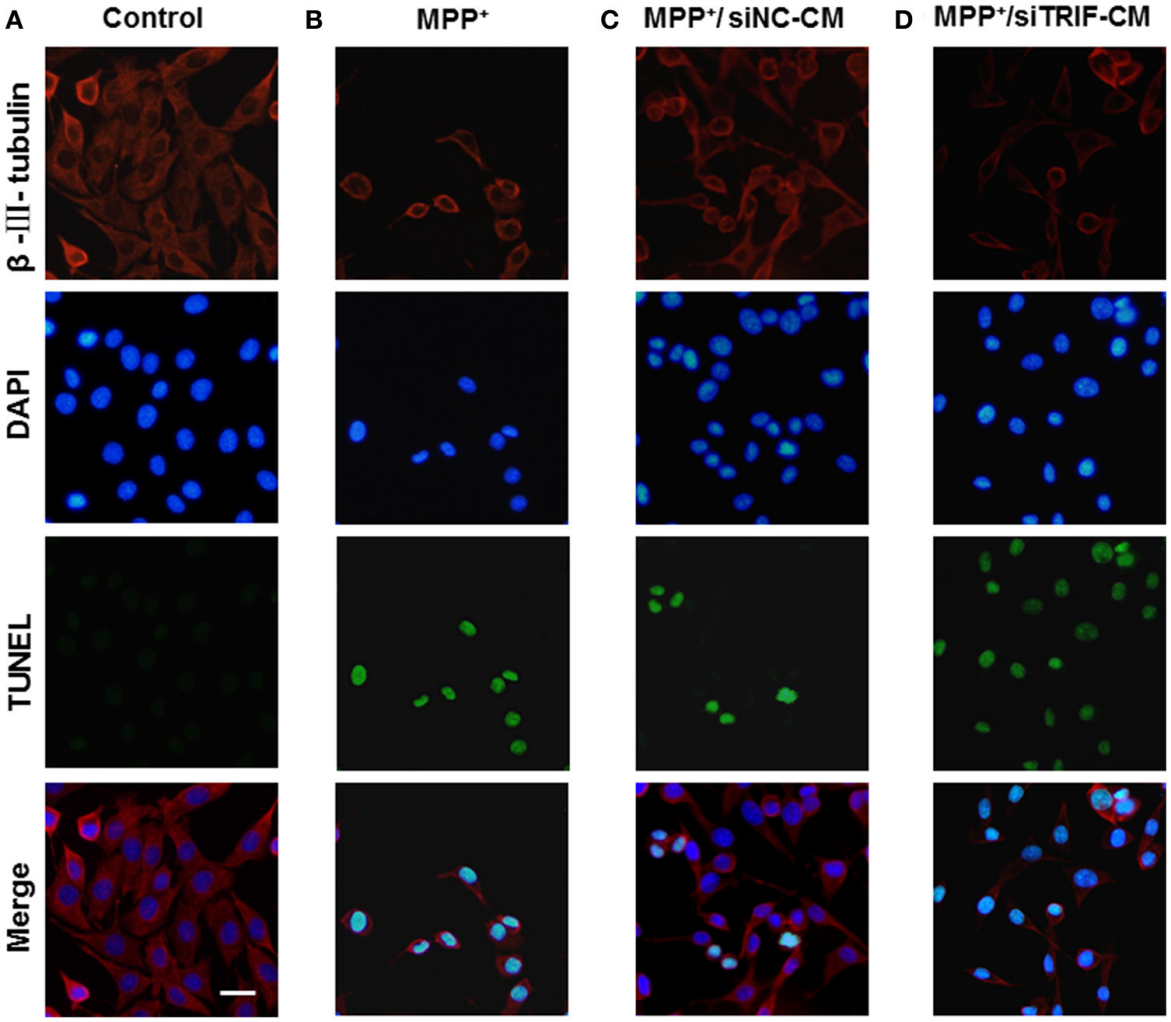
**Apoptosis of MN9D cells treated with BV2 cell-conditioned medium by TUNEL detectioin**. **(A)** Control group, MN9D cells treated with BV2 cell medium for 24 h. **(B)** MPP+ group (200 μM), MPP+-treated MN9D cells cultured with naive BV2 cell medium. **(C)** MPP+/siNC-CM group. MPP+ (200 μM) treated MN9D cells cultured with conditioned medium containing negative control siRNA (siNC-CM) in BV2 cells. **(D)** MPP+/siTRIF-CM group. MPP+ (200 μM) treated MN9D cells cultured with BV2 cell conditioned medium containing siTRIF (siTRIF-CM).

**Figure 8:**
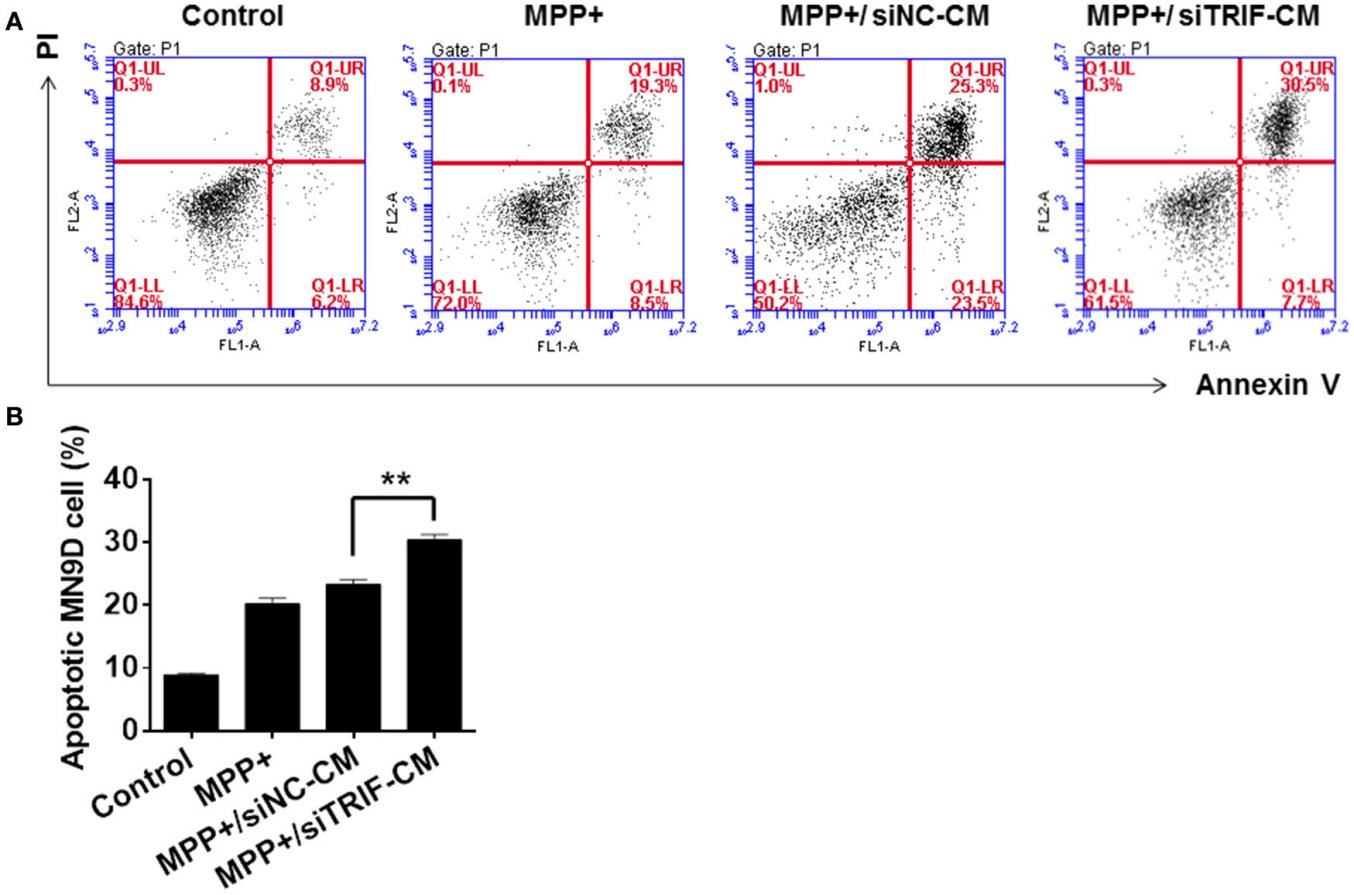
**Apoptosis of MN9D cells treated with BV2 cell conditioned medium by PI/Annexin V detection. (A)** Apoptosis of control, MPP+, MPP+/siNC-CM, and MPP+/siTRIF-CM groups of MN9D cells by flow cytometry detection with PI/Annexin V staining. 8.9% PI/Annexin V staining of cells in the control group; 25.3% PI/Annexin V staining of cells in the MPP+ group; 19.3% PI/Annexin V staining of cells in the MPP+/siNC-CM group; 30.5% PI/Annexin V staining of cells in the MPP+/siTRIF-CM group. **(B)** Quantification of PI/Annexin V double staining of cells in the control, MPP+, MPP+/siNC-CM, and MPP+/siTRIF-CM groups. Similar number of apoptotic cells in MPP+ and MPP+/siTRIF-CM groups. But significant differences between MPP+/siNC-CM and MPP+/siTRIF-CM groups of MN9D cells. NC, negative control. CM, conditioned medium. *n* = 3, mean ± SEM, ^**^p < 0.01.

As we have found the M1/M2 paradigm of microglia was triggered by MPP+-injured MN9D cells, the microglial cells can be polarized by the release of different typical pro/anti-inflammatory factors. However, we did not know how MN9D cell apoptosis was affected by microglial polarization. In this section, the co-cultured MN9D cells and BV2 cells were studied especially when siTRIF was applied. As a result, by βIII-tubulin and TUNEL dual labeling, the number of apoptotic MN9D cells in the MPP+ group accounts for 24 ± 5% of the total number of MN9D cells, however, the number increased drastically to 35 ± 3% when co-cultured with BV2 cells. And the number of apoptotic MN9D cells reached over 48 ± 4% with siTRIF treatment (Figures 7A–D, *n* = 3, *p* < 0.05). The exact number of apoptotic cells was quantified and analyzed by flow cytometry with Annexin-V-FITC /PI double staining (Figure 8, *n* = 3, *p* < 0.05). The siTRIF/MPP+ group showed the highest number of apoptotic MN9D cells. Pro-inflammatory stimuli and stress conditions increase the DA neuron apoptosis *in vivo* (Glass et al., 2010). In this section, we have found that the siTRIF treatment of BV2 cells may aggravate the apoptosis of MPP+-treated MN9D cells. Here, TRIF may play a protective role in MN9D cell apoptosis.

**Figure 9:**
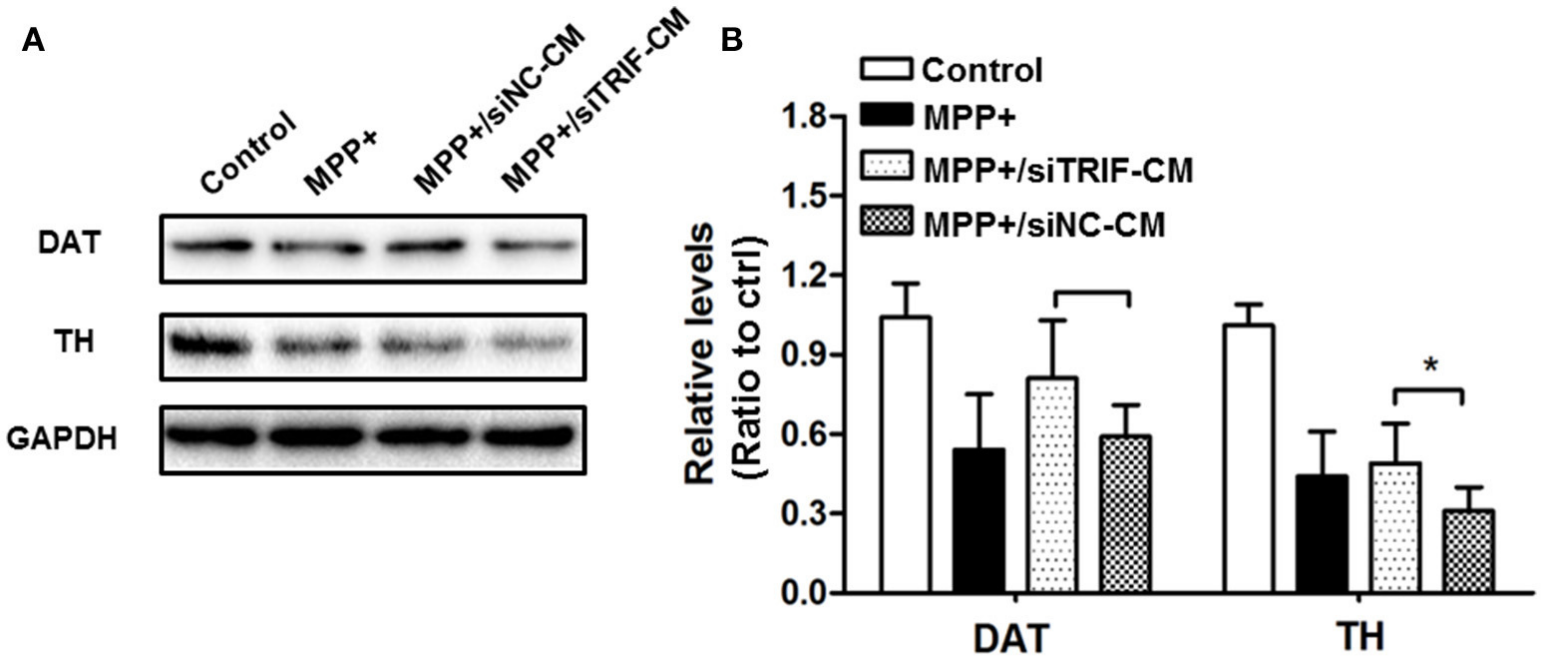
**Effect of TRIF deficiency in BV2 cells on the expression of DAT/TH protein in MN9D cells in a co-culture system**. **(A)** The expression levels of DAT and TH protein in MN9D cells in co-culture with siTRIF-treated BV2 cells. The expressions of DAT and TH decreased in MPP+, MPP+/siNC-CM, and MPP+/siTRIF-CM groups compared with control groups. **(B)** Quantification of the expression of DAT and TH in different groups. Significant differences were found between MPP+/siNC-CM, and MPP+/siTRIF-CM groups, respectively. *n* = 3, mean ± SEM, ^*^p < 0.05.

MPP+ is the conversion product of the neurotoxin MPTP in the brain, which is a high-affinity substrate for DAT as well as for norepinephrine and serotonin transporters (Javitch et al., 1985). DAT is the required step for MPTP neurotoxicity as evidenced by the fact that antagonist inhibition or genetic ablation of DAT prevents MPTP-induced dopaminergic neurodegeneration (Uhl et al., 1985). As TH functioned as a rate-limiting step in catalyzing the formation of L-DOPA, PD can be also considered as a TH-deficiency syndrome (Haavik and Toska, 1998). In this study, DAT and TH were detected by Western blot to verify the TRIF function in regulating MN9D cell dopaminergic function. As a result, it was indicated that the siTRIF treated BV2 cells affect the DAT and TH expression by MPP+-treated MN9D cells (Figure 9, *n* = 3, *p* < 0.05). There is a significant difference between the siNC-CM group and the siTRIF-CM group, which suggests TRIF suppression may aggravate MN9D cell dopaminergic functions.

## Discussion

In this study, we present evidence that TRIF is crucial for the microglial M1/M2 paradigm. Suppression of TRIF may switch microglia from the beneficial M2 phenotype to the harmful M1 phenotype and finally aggravates MPP+-induced MN9D cells apoptosis.

In a MPTP-induced neurodegenerative model, we firstly showed dramatically decreased concentrations of DA-related metabolites in *Trif* ^−/−^ mice which suggests that TRIF may play a protective role in MPTP-induced DA neuron degeneration. To confirm the phenotype, we labeled DA neurons with TH antibody in the SNpc region. Consistant with the HPLC-EC results, less TH-positive cells resided in the SNpc in *Trif* ^−/−^ mice (Figure 2F). Even with poly(I:C) stimulation and in rescue experiments, the number of DA neurons was still less compared with the WT group (Figures 2C,G).

Poly(I:C) is the agonist of TLR3, and TRIF is the sole adaptor or TLR3, which suggests that poly(I:C) also can be considered as an indirect agonist of TRIF (Doyle et al., 2002) which may selectively activate the IRF-3 signaling pathway. Our results indicate that the TRIF-IRF3 signaling pathway can be inhibited by siTRIF in the BV2 cell line. BV2, an immortalized murine microglial cell line, has been used commonly as a substitute for primary cultured microglial cells (Bocchini et al., 1992; Henn et al., 2009). The BV2 cell line has similarity with primary microglial cells with respect to the transcriptome (480 genes) and proteome analyses when stimulated by LPS (Henn et al., 2009). Although some other studies described the TLR-MyD88-NFκB reactions in BV2 cells in different immunological mechanisms and behaviors, and BV2 could be activated by poly(I:C) (Nguyen et al., 2013; Guo et al., 2015), we found for the first time that the suppression of TRIF results in inhibition of IRF-3 phosphorylation and downstream IFN-β release in BV2 cells, which suggests that BV2 is an ideal cell model for the TRIF study in this project. In our previous study, the deletion of the TRIF gene inhibited the microglial migration *in vitro* when co-cultured with retinal ganglion cells (RGCs; Lin et al., 2012a). Interestingly, the present results revealed that inhibition of TRIF by siRNA increases the migration ability of BV2 cells even with MPP+ and poly(I:C) stimulation, which supported the putative role of TRIF in microglial migration.

Increasingly, it has been demonstrated that microglial activation can be classified into two major phenotypes, the M1 detrimental phenotype and the M2 anti-inflammatory phenotype which promotes tissue and cell repair (Hanisch and Kettenmann, 2007; Kim et al., 2013). The M1/M2 paradigm markers of microglia and microglial polarity may change through the pathologic process. Here, we present evidence that the classical microglial M1/M2 markers changed depending on LPS and IL-4 stimulation (Figure 5A). However, in TRIF knock down experiments the polarities of microglia changed when different typical markers decreased, i.e., TNF-α, IL-6, CD86, CD206, and Arg1 (Figures 5B–D,G). Interestingly, the changed markers are indicators of both M1 and M2 microglial polarization, which suggests the knockdown of TRIF may regulate BV2 into either detrimental or beneficial roles. However, M1 and M2 phenotypes of microglial cells may simultaneously coexist (Fenn et al., 2014) as the mRNAs of inflammatory factors do not often change sharply along the spectrum of the M1/M2 paradigm. Furthermore, the dynamic function of the microglial M1/M2 paradigm is more complicated than the change of a limited number of polarity-related genes.

The mechanism of microglial dynamic polarization is still unclear, in which M1 and M2 microglia are derived from different phenotype shifting states, or subpopulations may have different functions, although the heterogeneous mixed glial cells can be separated (Hu et al., 2007; Shimizu et al., 2008; Chhor et al., 2013). M1 and M2 microglia can be induced phenotypically and functionally in response to external factors *in vitro* (Tanaka et al., 2015), whereas the similar microglial polarization was observed *in vivo*, in which M2 microglia were transient (Kigerl et al., 2009). Furthermore, M2 cell polarization is essential for efficient remyelination of CNS regeneration *in vivo* (Miron et al., 2013), which suggests that M2 microglia exist and may contribute to the CNS protection and improved neuronal function (Liu et al., 2012). Recently, Ransohoff summarized the significance of the microglial M1 and M2 phenotype. It was indicated that based on the RNA-seq analysis and unbiased approaches such as genome-wide transcriptomics and epigenomics, researchers found microglia will beyond either M1 or M2 transcriptomes under specific physiological or stress conditions (Ransohoff, 2016). Therefore, the microglial paradigm is more complicated and depends on specific microglial conditions used in external and internal strategies and more research work in the field is needed in the future.

In an MPP+-induced *in vitro* neurodegenerative model, the migration assay showed that the conditioned medium (CM) from MPP+-treated mesencephalic cultures was enough to attract microglia at early and late phase of neuronal damage (Kim et al., 2012). In the present study, the BV2 and MN9D cells were co-cultured to explore the influence of TRIF knockdown on the regulation of M1/M2 microglial polarization in an *in vitro* model. The MN9D cells were firstly treated with MPP+ and the medium was replaced when co-cultured with BV2 cells. Therefore, the BV2 cell activation and the markers' spectrum variation depended on the injured MN9D cells. The downstream targets of TRIF are IRF3, IRF7, and NF-κB (Hiscott et al., 2006), which activate the pro- and anti-inflammatory genes. To the best of our knowledge, the current divergent results showed for the first time the complexity of the M1/M2 microglial paradigm in an *in vitro* PD model. It may be partly due to the limited microglial M1/M2 marker spectrum we have screened. Downstream molecules need to be investigated in more detail. Furthermore, the effect of BV2 cells on MN9D cells was investigated by means of apoptosis detection. The largest number of apoptotic MN9D cells was found in the TRIF knockdown group (Figure 7D) and the results were also verified by flow cytometry (Figures 8A,B) which interestingly suggested that the suppression of TRIF aggravates MPP+-treated MN9D cell apoptosis.

Our results demonstrate an impaired M1/M2 polarization in BV2 cells by TRIF suppression, and as a result the co-cultured MN9D cells exhibit more apoptosis. Upon persistent and overabundant inflammation triggered by M1 microglial cells, additional inflammatory cytokines are produced and a loop is generated that in turn induces further inflammation and maintains the microglial M1 phenotype. As a result, the loop-skewed M1 microglia impaired phagocytic function and is neurotoxic in Alzheimer's disease and multiple sclerosis (Cherry et al., 2014). In addition, under certain pathological conditions the M2 microglia may unusually enhance disease development (Vaknin et al., 2011; Cherry et al., 2014). However, this is the first study that discovered the relationship between the microglial M1/M2 phenotype and TRIF regulation. As TRIF is a downstream adaptor of TLR3 and TLR4, a number of well-studied antagonists and agonists can be used to directly regulate the level of TRIF expression in CNS. Thus, this study provides some helpful hints for further research in neuropharmacological methods and molecules that may regulate the PD process. The detailed mechanism needs to be elucidated by further experiments and screening the spectrum of inflammatory factors.

## Author contributions

BS, SRL, and SL designed the experiment; SL contributed to data acquisition, analysis and interpretation, and wrote the manuscript. MS, YD, HXZ, HH, MY, XY, YW, LR, JP, JS, and HLZ performed the experiments; including realtime PCR, Western blot, TUNEL, cell culture, flow cytometry, and cell migration test. All authors have read and approved the final manuscript.

## Funding

This work was supported by the National Nature Science Foundation of China (31371215, 31540032, and 81200926), the Fund of Development and Regeneration Key Laboratory of Sichuan Province (SYS15-002, SYS14-001, and SYS11-002) and the fund of Major Project of Education Department in Sichuan (14ZA0222).

### Conflict of interest statement

The authors declare that the research was conducted in the absence of any commercial or financial relationships that could be construed as a potential conflict of interest.

## References

[B1] Abercrombie M. (1946). Estimation of nuclear population from microtome sections. Anat. Rec. 94, 239–247. 10.1002/ar.1090940210 21015608

[B2] Barcia C. Ros C. M. Ros-Bernal F. Gomez A. Annese V. Carrillo-de Sauvage M. A.. (2013). Persistent phagocytic characteristics of microglia in the substantia nigra of long-term Parkinsonian macaques. J. Neuroimmunol. 261, 60–66. 10.1016/j.jneuroim.2013.05.001 23759319

[B3] Block M. L. Zecca L. Hong J. S. (2007). Microglia-mediated neurotoxicity: uncovering the molecular mechanisms. Nat. Rev. Neurosci. 8, 57–69. 10.1038/nrn2038 17180163

[B4] Bocchini V. Mazzolla R. Barluzzi R. Blasi E. Sick P. Kettenmann H. (1992). An immortalized cell line expresses properties of activated microglial cells. J. Neurosci. Res. 31, 616–621. 10.1002/jnr.490310405 1578513

[B5] Bruni D. Sebastia J. Dunne S. Schroder M. Butler M. P. (2013). A novel IRAK1-IKKepsilon signaling axis limits the activation of TAK1-IKKbeta downstream of TLR3. J. Immunol. 190, 2844–2856. 10.4049/jimmunol.1202042 23396947

[B6] Cherry J. D. Olschowka J. A. O'Banion M. K. (2014). Neuroinflammation and M2 microglia: the good, the bad, and the inflamed. J. Neuroinflamm. 11:98. 10.1186/1742-2094-11-98 24889886 PMC4060849

[B7] Chhor V. Le Charpentier T. Lebon S. Ore M. V. Celador I. L. Josserand J.. (2013). Characterization of phenotype markers and neuronotoxic potential of polarised primary microglia *in vitro*. Brain Behav. Immun. 32, 70–85. 10.1016/j.bbi.2013.02.005 23454862 PMC3694309

[B8] Colton C. A. (2009). Heterogeneity of microglial activation in the innate immune response in the brain. J. Neuroimmune Pharmacol. 4, 399–418. 10.1007/s11481-009-9164-4 19655259 PMC2773116

[B9] Colton C. Wilcock D. M. (2010). Assessing activation states in microglia. CNS Neurol. Disord. Drug Targets 9, 174–191. 10.2174/187152710791012053 20205642

[B10] Crocker S. J. Smith P. D. Jackson-Lewis V. Lamba W. R. Hayley S. P. Grimm E.. (2003). Inhibition of calpains prevents neuronal and behavioral deficits in an MPTP mouse model of Parkinson's disease. J. Neurosci. 23, 4081–4091. 12764095 10.1523/JNEUROSCI.23-10-04081.2003 PMC6741113

[B11] Daffis S. Samuel M. A. Suthar M. S. Gale M. Jr. Diamond M. S. (2008). Toll-like receptor 3 has a protective role against West Nile virus infection. J. Virol. 82, 10349–10358. 10.1128/JVI.00935-08 18715906 PMC2573187

[B12] Dauer W. Przedborski S. (2003). Parkinson's disease: mechanisms and models. Neuron 39, 889–909. 10.1016/S0896-6273(03)00568-3 12971891

[B13] David S. Kroner A. (2011). Repertoire of microglial and macrophage responses after spinal cord injury. Nat. Rev. Neurosci. 12, 388–399. 10.1038/nrn3053 21673720

[B14] Doring A. Yong V. W. (2011). The good, the bad and the ugly. Macrophages/microglia with a focus on myelin repair. Front. Biosci. 3, 846–856. 10.2741/191 21622236

[B15] Doyle S. Vaidya S. O'Connell R. Dadgostar H. Dempsey P. Wu T.. (2002). IRF3 mediates a TLR3/TLR4-specific antiviral gene program. Immunity 17, 251–263. 10.1016/S1074-7613(02)00390-4 12354379

[B16] Fenn A. M. Hall J. C. Gensel J. C. Popovich P. G. Godbout J. P. (2014). IL-4 signaling drives a unique arginase^+^/IL-1β^+^ microglia phenotype and recruits macrophages to the inflammatory CNS: consequences of age-related deficits in IL-4Rα after traumatic spinal cord injury. J. Neurosci. 34, 8904–8917. 10.1523/JNEUROSCI.1146-14.2014 24966389 PMC4069360

[B17] Ferrari C. C. Tarelli R. (2011). Parkinson's disease and systemic inflammation. Parkinsons Dis. 2011:436813. 10.4061/2011/436813 21403862 PMC3049348

[B18] Gao H. M. Hong J. S. (2008). Why neurodegenerative diseases are progressive: uncontrolled inflammation drives disease progression. Trends Immunol. 29, 357–365. 10.1016/j.it.2008.05.002 18599350 PMC4794280

[B19] Gerhard A. Pavese N. Hotton G. Turkheimer F. Es M. Hammers A. (2006). *In vivo* imaging of microglial activation with [11C]®-PK11195 PET in idiopathic Parkinson's disease. Neurobiol. Dis. 21, 404–412. 10.1016/j.nbd.2005.08.002 16182554

[B20] Glass C. K. Saijo K. Winner B. Marchetto M. C. Gage F. H. (2010). Mechanisms underlying inflammation in neurodegeneration. Cell 140, 918–934. 10.1016/j.cell.2010.02.016 20303880 PMC2873093

[B21] Guo Y. J. Luo T. Wu F. Mei Y. W. Peng J. Liu H.. (2015). Involvement of TLR2 and TLR9 in the anti-inflammatory effects of chlorogenic acid in HSV-1-infected microglia. Life Sci. 127, 12–18. 10.1016/j.lfs.2015.01.036 25744394

[B22] Haavik J. Toska K. (1998). Tyrosine hydroxylase and Parkinson's disease. Mol. Neurobiol. 16, 285–309. 10.1007/BF02741387 9626667

[B23] Hanisch U. K. Kettenmann H. (2007). Microglia: active sensor and versatile effector cells in the normal and pathologic brain. Nat. Neurosci. 10, 1387–1394. 10.1038/nn1997 17965659

[B24] Henn A. Lund S. Hedtjarn M. Schrattenholz A. Porzgen P. Leist M. (2009). The suitability of BV2 cells as alternative model system for primary microglia cultures or for animal experiments examining brain inflammation. Altex 26, 83–94. 10.14573/altex.2009.2.83 19565166

[B25] Hiscott J. Nguyen T. L. Arguello M. Nakhaei P. Paz S. (2006). Manipulation of the nuclear factor-kappaB pathway and the innate immune response by viruses. Oncogene 25, 6844–6867. 10.1038/sj.onc.1209941 17072332 PMC7100320

[B26] Hu X. Chen J. Wang L. Ivashkiv L. B. (2007). Crosstalk among Jak-STAT, Toll-like receptor, and ITAM-dependent pathways in macrophage activation. J. Leukoc. Biol. 82, 237–243. 10.1189/jlb.1206763 17502339

[B27] Javitch J. A. D'Amato R. J. Strittmatter S. M. Snyder S. H. (1985). Parkinsonism-inducing neurotoxin, N-methyl-4-phenyl-1,2,3,6 -tetrahydropyridine: uptake of the metabolite N-methyl-4-phenylpyridine by dopamine neurons explains selective toxicity. Proc. Natl. Acad. Sci. U.S.A. 82, 2173–2177. 10.1073/pnas.82.7.2173 3872460 PMC397515

[B28] Kigerl K. A. Gensel J. C. Ankeny D. P. Alexander J. K. Donnelly D. J. Popovich P. G. (2009). Identification of two distinct macrophage subsets with divergent effects causing either neurotoxicity or regeneration in the injured mouse spinal cord. J. Neurosci. 29, 13435–13444. 10.1523/JNEUROSCI.3257-09.2009 19864556 PMC2788152

[B29] Kim C. Ho D. H. Suk J. E. You S. Michael S. Kang J.. (2013). Neuron-released oligomeric alpha-synuclein is an endogenous agonist of TLR2 for paracrine activation of microglia. Nat. Commun. 4:1562. 10.1038/ncomms2534 23463005 PMC4089961

[B30] Kim H. Park J. H. Kim K. (2012). Lipid-like components released from degenerating dopaminergic neurons trigger the dynamic migration of microglia. Biochem. Biophys. Res. Commun. 426, 18–25. 10.1016/j.bbrc.2012.07.167 22898047

[B31] LaVoie M. J. Card J. P. Hastings T. G. (2004). Microglial activation precedes dopamine terminal pathology in methamphetamine-induced neurotoxicity. Exp. Neurol. 187, 47–57. 10.1016/j.expneurol.2004.01.010 15081587

[B32] Le W. Rowe D. Xie W. Ortiz I. He Y. Appel S. H. (2001). Microglial activation and dopaminergic cell injury: an *in vitro* model relevant to Parkinson's disease. J. Neurosci. 21, 8447–8455. 11606633 10.1523/JNEUROSCI.21-21-08447.2001 PMC6762816

[B33] Liang Y. Li S. Guo Q. Zhang Y. Wen C. Zou Q. (2007). Complement 3-deficient mice are not protected against MPTP-induced dopaminergic neurotoxicity. Brain Res. 1178, 132–140. 10.1016/j.brainres.2007.08.033 17900537

[B34] Liang Y. Li S. Wen C. Zhang Y. Guo Q. Wang H.. (2008). Intrastriatal injection of colchicine induces striatonigral degeneration in mice. J. Neurochem. 106, 1815–1827. 10.1111/j.1471-4159.2008.05526.x 18564367

[B35] Liang Y. Li S. Zou Q. Su B. (2006). Potential neuroprotective effect of low dose whole-body gamma-irradiation against 1-methyl-4-phenyl-1,2,3,6-tetrahydropyridine (MPTP)-induced dopaminergic toxicity in C57 mice. Neurosci. Lett. 400, 213–217. 10.1016/j.neulet.2006.02.061 16540245

[B36] Liao B. Zhao W. Beers D. R. Henkel J. S. Appel S. H. (2012). Transformation from a neuroprotective to a neurotoxic microglial phenotype in a mouse model of ALS. Exp. Neurol. 237, 147–152. 10.1016/j.expneurol.2012.06.011 22735487 PMC4126417

[B37] Lin S. Liang Y. Zhang J. Bian C. Zhou H. Guo Q.. (2012a). Microglial TIR-domain-containing adapter-inducing interferon-beta (TRIF) deficiency promotes retinal ganglion cell survival and axon regeneration via nuclear factor-kappaB. J. Neuroinflamm. 9:39. 10.1186/1742-2094-9-39 22361049 PMC3471332

[B38] Lin S. Yin Q. Zhong Q. Lv F. L. Zhou Y. Li J. Q.. (2012b). Heme activates TLR4-mediated inflammatory injury via MyD88/TRIF signaling pathway in intracerebral hemorrhage. J. Neuroinflamm. 9:46. 10.1186/1742-2094-9-46 22394415 PMC3344687

[B39] Liu S. Cai X. Wu J. Cong Q. Chen X. Li T.. (2015). Phosphorylation of innate immune adaptor proteins MAVS, STING, and TRIF induces IRF3 activation. Science 347:aaa2630. 10.1126/science.aaa2630 25636800

[B40] Liu S. Liu Y. Hao W. Wolf L. Kiliaan A. J. Penke B.. (2012). TLR2 is a primary receptor for Alzheimer's amyloid beta peptide to trigger neuroinflammatory activation. J. Immunol. 188, 1098–1107. 10.4049/jimmunol.1101121 22198949

[B41] Miron V. E. Boyd A. Zhao J. W. Yuen T. J. Ruckh J. M. Shadrach J. L.. (2013). M2 microglia and macrophages drive oligodendrocyte differentiation during CNS remyelination. Nat. Neurosci. 16, 1211–1218. 10.1038/nn.3469 23872599 PMC3977045

[B42] Nguyen T. T. Kim Y. M. Kim T. D. Le O. T. Kim J. J. Kang H. C.. (2013). Phosphatidylinositol 4-phosphate 5-kinase alpha facilitates Toll-like receptor 4-mediated microglial inflammation through regulation of the Toll/interleukin-1 receptor domain-containing adaptor protein (TIRAP) location. J. Biol. Chem. 288, 5645–5659. 10.1074/jbc.M112.410126 23297396 PMC3581370

[B43] Ouchi Y. Yoshikawa E. Sekine Y. Futatsubashi M. Kanno T. Ogusu T.. (2005). Microglial activation and dopamine terminal loss in early Parkinson's disease. Ann. Neurol. 57, 168–175. 10.1002/ana.20338 15668962

[B44] Patel A. K. Hackam A. S. (2013). Toll-like receptor 3 (TLR3) protects retinal pigmented epithelium (RPE) cells from oxidative stress through a STAT3-dependent mechanism. Mol. Immunol. 54, 122–131. 10.1016/j.molimm.2012.11.005 23267850 PMC3640317

[B45] Ponomarev E. D. Maresz K. Tan Y. Dittel B. N. (2007). CNS-derived interleukin-4 is essential for the regulation of autoimmune inflammation and induces a state of alternative activation in microglial cells. J. Neurosci. 27, 10714–10721. 10.1523/JNEUROSCI.1922-07.2007 17913905 PMC6672829

[B46] Ransohoff R. M. (2016). A polarizing question: do M1 and M2 microglia exist? Nat. Neurosci. 19, 987–991. 10.1038/nn.4338 27459405

[B47] Sharma S. tenOever B. R. Grandvaux N. Zhou G. P. Lin R. Hiscott J. (2003). Triggering the interferon antiviral response through an IKK-related pathway. Science 300, 1148–1151. 10.1126/science.1081315 12702806

[B48] Shimizu E. Kawahara K. Kajizono M. Sawada M. Nakayama H. (2008). IL-4-induced selective clearance of oligomeric beta-amyloid peptide(1-42) by rat primary type 2 microglia. J. Immunol. 181, 6503–6513. 10.4049/jimmunol.181.9.6503 18941241

[B49] Stridh L. Mottahedin A. Johansson M. E. Valdez R. C. Northington F. Wang X.. (2013). Toll-like receptor-3 activation increases the vulnerability of the neonatal brain to hypoxia-ischemia. J. Neurosci. 33, 12041–12051. 10.1523/JNEUROSCI.0673-13.2013 23864690 PMC3713735

[B50] Tanaka T. Murakami K. Bando Y. Yoshida S. (2015). Interferon regulatory factor 7 participates in the M1-like microglial polarization switch. Glia 63, 595–610. 10.1002/glia.22770 25422089

[B51] Uhl G. R. Javitch J. A. Snyder S. H. (1985). Normal MPTP binding in parkinsonian substantial nigra: evidence for extraneuronal toxin conversion in human brain. Lancet 1, 956–957. 10.1016/S0140-6736(85)91729-5 2859415

[B52] Vaknin I. Kunis G. Miller O. Butovsky O. Bukshpan S. Beers D. R.. (2011). Excess circulating alternatively activated myeloid (M2) cells accelerate ALS progression while inhibiting experimental autoimmune encephalomyelitis. PLoS ONE 6:e26921. 10.1371/journal.pone.0026921 22073221 PMC3207825

[B53] Wang S. Chu C. H. Stewart T. Ginghina C. Wang Y. Nie H.. (2015). alpha-Synuclein, a chemoattractant, directs microglial migration via H_2_O_2_-dependent Lyn phosphorylation. Proc. Natl. Acad. Sci. U.S.A. 112, E1926–E1935. 10.1073/pnas.1417883112 25825709 PMC4403145

[B54] Yamamoto M. Sato S. Hemmi H. Hoshino K. Kaisho T. Sanjo H.. (2003). Role of adaptor TRIF in the MyD88-independent toll-like receptor signaling pathway. Science 301, 640–643. 10.1126/science.1087262 12855817

[B55] Yamamoto M. Sato S. Mori K. Hoshino K. Takeuchi O. Takeda K.. (2002). Cutting edge: a novel Toll/IL-1 receptor domain-containing adapter that preferentially activates the IFN-beta promoter in the Toll-like receptor signaling. J. Immunol. 169, 6668–6672. 10.4049/jimmunol.169.12.6668 12471095

